# How to show that a new imaging method can replace a standard method, when no reference standard is available?

**DOI:** 10.1007/s00330-021-08325-7

**Published:** 2021-11-18

**Authors:** Patrick Omoumi, Nancy A. Obuchowski

**Affiliations:** 1 Department of Radiology, Lausanne University Hospital and University of Lausanne, Rue du Bugnon 46, 1011 Lausanne, Switzerland; 2 Quantitative Health Sciences, The Cleveland Clinic Foundation, Cleveland, OH USA

Assessing the performance of a new diagnostic method is a common problem in radiology. With technical advances impacting image acquisition and post-processing, including applications of artificial intelligence, newer ways to perform imaging have emerged and need to be compared to the methods of reference.

Practical examples include low-dose CT examinations providing similar image quality as standard dose acquisitions thanks to improved image reconstruction [1–3], or accelerated MRI protocols by using novel image acquisition or image reconstruction methods [4, 5].

While the assessment of image quality and phantom studies might be the first steps of the evaluation, the diagnostic performance of the new imaging method should eventually be compared to that of the old method, typically using a reference standard such as surgery. The statistical methods for this type of analysis are well established [6, 7].

However, more often than not, there is no suitable or convenient reference standard against which the performance of the new diagnostic method can be tested. An example of this is imaging of the degenerative spine for which a surgical correlation is often missing. When performed, surgery only provides a limited amount of information compared to the extensive assessment that can be done by imaging (for example, no assessment of bone marrow can usually be done at spine surgery).

In such situations, investigators have tried a variety of approaches to quantify performance of the new method relative to the current one, such as reporting accuracy using the current test as the reference standard, assessing correlation of the new test’s findings with the current test, estimating intra- or inter-reader agreement of the new and existing tests, and testing for lack of significant differences between the findings of the tests.

These approaches, however, can provide misleading results. Alternatively, interchangeability is a statistical method to assess whether a new diagnostic method can replace a conventional method when there is no reference standard available. To illustrate, let us consider four studies on degenerative spine MRI taken from the recent literature [8–11].

These four studies aimed to show that MRI of the degenerative spine in the sagittal plane may be limited to a fast spin echo/turbo spin echo (FSE/TSE) Dixon fluid-sensitive sequence, with no need to perform additional T1-weighted (T1w) sequences. While the conclusions were the same, the approaches used were different, some being potentially misleading.

Sollmann et al tested for lack of significant differences between the number of abnormalities detected on the new protocol including fat-only (FO), in-phase (IP), and water-only (WO) images derived from a FSE/TSE Dixon T2-weighted sequence and the standard protocol (T1w, IP, WO images) [9]. However, this approach focuses on pooled results rather than individual subject results and suffers from low power to detect small but important differences.

Saiffudin et al studied the agreement between the new and standard protocols with different readers, and compared the inter-reader agreement obtained with the two protocols. This approach may also be misleading in various ways. First, poor inter-technique agreement may be due to poor intra-reader agreement for variables which are subjectively graded, thereby underestimating the potential for the new protocol to replace the standard one. In that study, kappa statistics for inter-protocol agreement were as low as 0.39 for some variables, but intra-reader agreement was not reported. Second, measures of intra- and inter-reader agreement, e.g., kappa statistic, tell us nothing about diagnostic performance.

In the study by Yang et al, diagnostic performance of the new protocol was quantified using the standard protocol as the reference standard. Because the new test often makes similar errors as the standard test, this approach usually leads to gross exaggeration of accuracy of the new protocol. Furthermore, when the new protocol disagrees with the standard one, it is misleading to assume that the standard protocol’s findings are always correct since it is not a true reference standard.

The first paper of the series, on the other hand, had used interchangeability to demonstrate that the new simplified protocol could replace the standard protocol [8]. Interchangeability is a statistical method that describes the effect of replacing the current test with the new test, without the need for a reference standard. The idea is to first quantify the ability of the current test to agree with itself (i.e., measure the inter-reader agreement where all readers use the current test), then compare this with the ability of the new test to agree with the current test. If the frequency of agreement and types of disagreements between new and current tests are similar to when the current test is compared with itself, then we conclude that the new test is interchangeable with the current test (Fig. 1). Interchangeability may be tested for both qualitative and quantitative data [12].

**Fig. 1:**
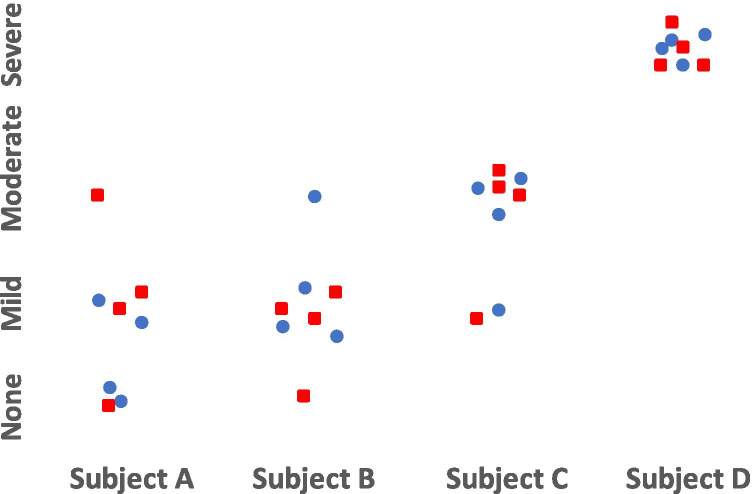
The findings of two protocols interpreted by four readers. The standard protocol findings are denoted by blue circles and the new protocol findings by red squares. Interchangeability is not achieved for subjects A and B because of additional discrepancies by the new protocol in the findings for subject A and differences in the types of findings for subject B. Interchangeability is achieved for subjects C and D, with similar frequency of discrepancies and types of findings for the two protocols

It is important that the definition of agreement and the maximum allowable difference between new and current vs. current with itself are defined a priori, and that the study is powered to detect these differences.

Apart from the initial study by Zanchi et al, there are several other examples of the use of interchangeability of imaging tests in the literature [8, 13, 14], as well as papers describing the statistical methods [12, 15, 16].

Finally, beyond these statistical considerations, it should be mentioned that interchangeability needs to be proven for all types of information that the standard method is supposed to provide in order for the new method to be able to replace it. For instance, in degenerative spine MRI, T1w images are also used for the assessment of bone marrow pathology. If the new protocol does not include T1w sequences, the information must be included in the other images [14]. Furthermore, the detailed acquisition parameters should also be specified in the study and taken into account when replacing a standard protocol by a new one. Indeed, what might be true for a certain sequence on a certain scanner might not be generalizable to all sequences on all scanners.

## Declarations

### Guarantor

The scientific guarantor of this publication is Dr Patrick Omoumi.

### Statistics and biometry

One of the authors is a statistician.

### Methodology

• Editorial
